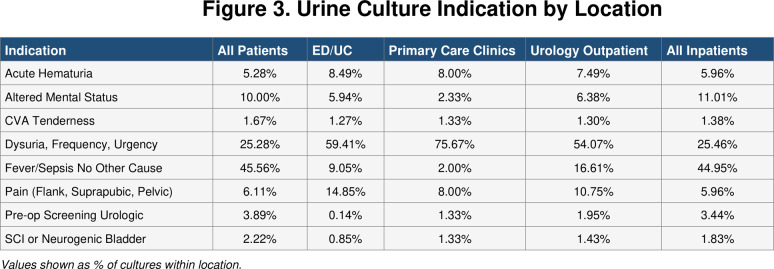# 193 Medical Complexity and Antibacterial Use: Evaluating the SAAR in Relation to Patient Population Acuity in Pennsylvania

**DOI:** 10.1017/ash.2026.10583

**Published:** 2026-06-23

**Authors:** Judith Strymish, Rebecca Madjarov, Dimitri Drekonja, School University of MN Medical, Erik Stensgard, Bobbie Masoud, Scott Connolly, Anna Chen, Katherine Linsenmeyer, Kalpana Gupta

**Affiliations:** 1 VA Med Ctr - West Roxbury; 2 VA Boston Healthcare System; 3 Minneapolis VA Health Care System; 4 Minneapolis VAHCS; 5 VA Boston and Boston University School of Medicine

## Abstract

**Background:** Inappropriate urine cultures without a clinical indication are sent frequently. Previous work has demonstrated that clinical decision support (CDS) requiring an indication for culture can reduce the number of unnecessary urine cultures. Our multi-campus VA facility implemented requirement of an indication for urine culture orders and evaluated the rate of urine cultures over time as well as the indications. **Methods:** We implemented a previously developed CDS menu across inpatient and outpatient services within VA Boston healthcare system. The CDS implementation required assistance and approval from our laboratory and clinical applications coordinators. Clinicians were notified about the change in ordering process through email and focused educational sessions. We captured rates of urine cultures by location and by indication and compared rates before and after implementation. A run-in period was allowed for full roll-out of the intervention, which was set at 98% of urine cultures being ordered through the CDS menu. This was a quality improvement initiative. **Result:** The study period included a 5-month pre-intervention period and a 5-month post-intervention period that followed a 2 month run-in period. A total of 2955 urine cultures were ordered preintervention compared with 2028 cultures postintervention. The average rate of monthly urine cultures decreased from 591/month to 406/month (P **Conclusion:** We demonstrated a significant reduction in urine culture orders using a clinical decision support intervention that required the ordering clinician to indicate a reason for the order. This intervention only required a targeted modification in our lab orders and was simple for clinicians to comply with given a drop down click menu. One limitation is that we did not capture clinical outcomes - but we did have continuous feedback from clinicians that did not indicate any adverse events, either in efficiency of practice or in outcomes among patients. This approach has been more successful at our hospital compared with other approaches that require system wide changes in urine culture processing or resulting. It has the added benefit of direct clinician education regarding appropriate urine culture indications in different settings.

**Figure:**
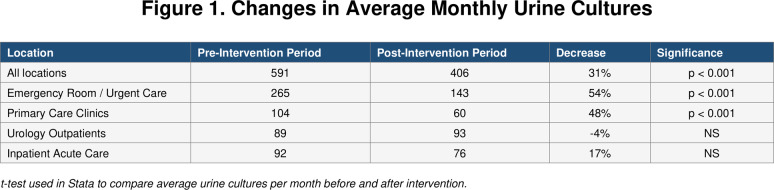

**Figure:**
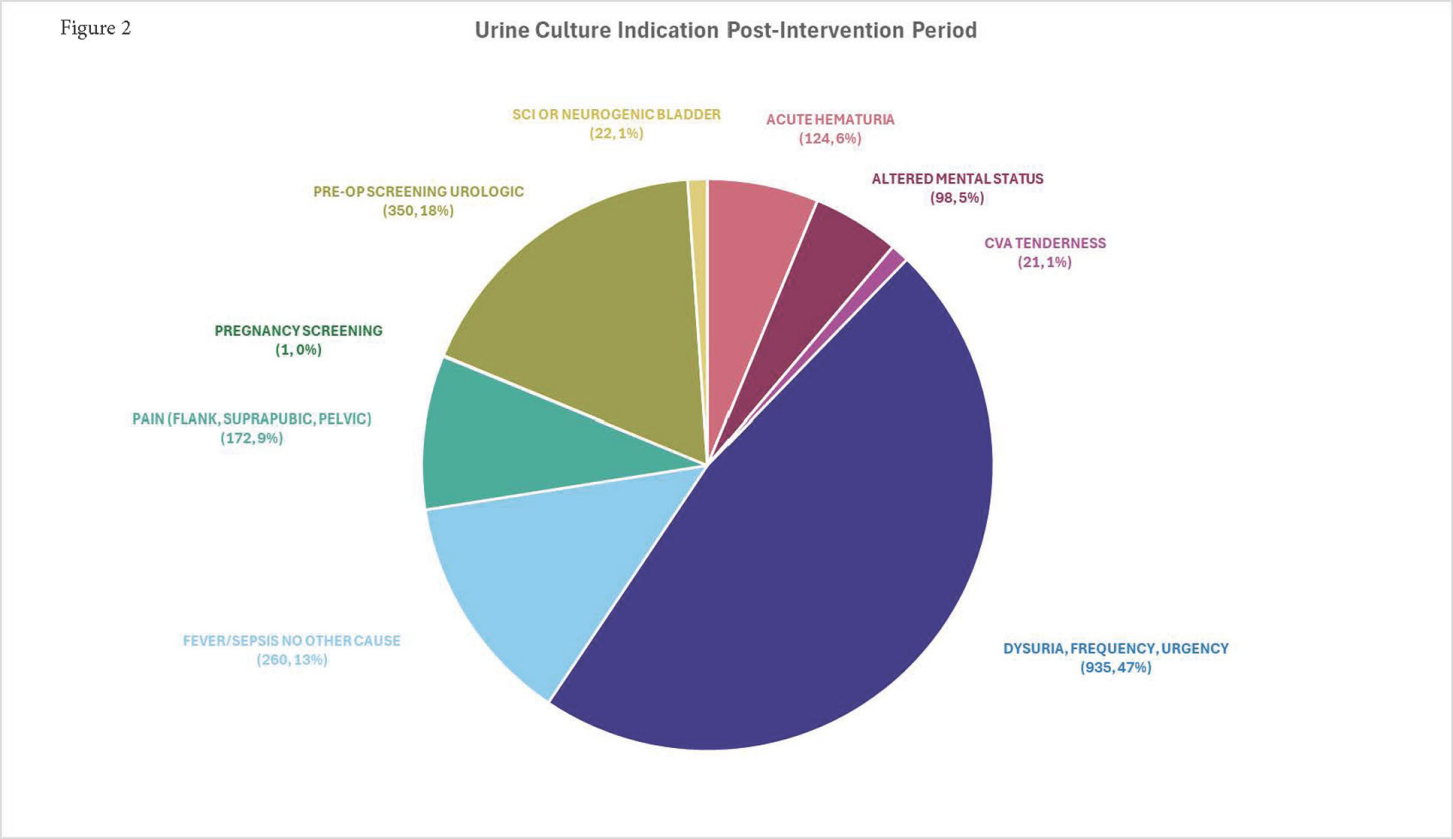

**Figure:**